# The Effects of Aromatherapy on Intensive Care Unit Patients' Stress and Sleep Quality: A Nonrandomised Controlled Trial

**DOI:** 10.1155/2017/2856592

**Published:** 2017-12-11

**Authors:** Eun Hee Cho, Mi-Young Lee, Myung-Haeng Hur

**Affiliations:** College of Nursing, Eulji University, Daejeon, Republic of Korea

## Abstract

**Background:**

Stress has both physiological and psychological effects and can negatively impact patients' treatment and recovery. We examined whether the aromatherapy alleviated patients' stress and improved their sleep quality and provided data that can be utilized in clinical settings.

**Methods:**

This was a nonrandomised controlled experimental study. Participants included lucid adult patients who were admitted to the intensive care unit and had spent more than two nights there. The experimental treatment required participants to engage in deep breathing with essential oils as part of the aromatherapy. The control group was instructed to go to sleep without receiving the lavender aroma oil.

**Results:**

The experimental group and control group showed a significant difference in perceived stress (*F* = 60.11, *p* < .001), objective stress index (*F* = 25.65, *p* < .001), systolic blood pressure (*F* = 9.09, *p* < .001), diastolic blood pressure (*F* = 2.47, *p* = .046), heart rate (*F* = 5.71, *p* < .001), and sleep quality (*F* = 109.46, *p* < .001).

**Conclusions:**

The results revealed that aromatherapy alleviated stress and improved sleep quality in intensive care unit patients after 2 days of the experimental treatment. These results demonstrate that aromatherapy affects stress and sleep quality, thus indicating its value in nursing interventions. This trial is registered with KCT0002344.

## 1. Introduction

Aside from the stress that accompanies illness, hospitalization is a stressful life event that brings about changes in one's daily life and the activities that they engage in. Consequently, patients often encounter psychological and social stress [1]. Stress manifests itself because of the interaction between one's internal propensities and external stimuli that is determined by their environment. This determines an individuals' coping response. Humans exhibit various symptoms in stressful situations, including an increase in physiological symptoms such as headaches and exhaustion; emotional symptoms such as anger, sadness, and helplessness; and behavioural symptoms such as crying and criticizing [2].

Most patients admitted into the intensive care unit (ICU) experience more severe symptoms and a higher likelihood of death. This increases their nursing needs and requires the patient to undergo continuous and intensive observation. The ICU is surrounded by medical teams and a variety of mechanical devices. The fear of being an ICU patient, uncertainty about the future, isolation from family, financial pressure, and exposure to an unfamiliar environment result in severe emotional imbalances in patients [3, 4]. In the ICU, a hospital's entire medical capacity is concentrated on providing patients with life-threatening illnesses with a chance at recovery. In the ICU environment, most patients experience stress due to anxiety about their prognosis; the unfamiliarity of the ICU and its treatment; limited visiting hours; the pressure of the inspection process; and cognitive, emotional, and behavioural stress depending on the dispositions of medical team members. This can cause the patient to resort to inappropriate coping methods [5]. Relatives of ICU patients report high levels of anxiety, depression, and feelings of panic, chaos, and a need for constant vigilance [4].

Psychological instability typically occurs when exhaustion starts to accumulate from stress, and the sympathetic nervous system, which maintains the body's state of equilibrium, is activated. This results in problems such as increased blood pressure, heightened tension, and sleep disorders [6]. It is unclear how stress affects sleep; however, the intimate temporal relationship between the stress of sleep architecture and the hypothalamo-pituitary-adrenal axis plays a vital role [7]. This axis and sympathetic nervous system activity are positively correlated with the total amount of rapid-eye-movement sleep [8].

Sleep is a basic human need, and maintaining good sleep quality is extremely important in preserving a healthy lifestyle [9]. Intensive care unit patients may result in problems such as decreased cognitive function, irritability, aggression, and disruptions in the sleep-wake cycle, which are associated with symptoms of disorientation and are reported to lead to the development of ICU syndrome [10]. There is a high correlation between stress and sleep quality; therefore, there is an urgent demand for nursing intervention to decrease stress and increase sleep quality in ICU patients with weakened immune systems [11].

Until now, stress studies addressed effect of inhalation of lavender essential oil on vital signs in open-heart surgery ICU [12], the effect of providing advanced information on the ICU environment and its impact on anxiety and environmental stress in open-heart surgery patients [4], and effect of inhalation aromatherapy with lavender essential oil on stress and vital signs in patients undergoing coronary artery bypass surgery [13].

Studies such as giving information as nursing interventions claim that these are partially effective for treating anxiety and stress. However, these studies were limited because they only focus on the effects of anxiety and stress on sleep. Studies on sleep quality in ICU patients have attempted to determine the phenomenon of sleep itself by examining sleep patterns, sleep disorder characteristics and factors [14], and factors related to sleep among ICU patients. However, there have been virtually no studies that attempt to improve sleep quality in these patients. ICU patients who are in a lucid state experience elevated levels of stress resulting from fear about survival due to their illness, concerns about their family and finances, limited movement, and the noise of the ICU. They also experience a loss in sleep quality. Because of the severity of their illness, however, patients are more interested in their condition than their basic need for quality sleep. A method to enhance sleep quality must be developed since a loss in sleep quality leads to increased stress. Nurses who have the most direct and continuous contact with ICU patients can play a key role in alleviating patients' stress and improving sleep quality.

Aromatherapy has recently gained traction as an alternative therapy. It perceives humans as holistic beings and focuses on their balance and harmony as components of nature [15]. Aromatherapy is a type of complementary alternative treatment that promotes physical, emotional, and psychological health by using the therapeutic elements of essential oils found in the flowers and leaves of various natural plants, stems, and roots [16]. Aromatherapy is a combination of aroma, treatment, and therapy that uses aroma essential oils to achieve balance in the patient by calming the mind, body, and spirit. It is also used in treatments to instil vitality [17]. In contrast to chemicals, aromatherapy is a relatively effective and safe treatment that does not accumulate in the body, but is discharged from the through the respiratory system, liver, and kidneys [18]. Aromatherapy is a noninvasive treatment that directly affects the brain, and individuals can self-administer the treatment regardless of time or place [19]. In addition, depending on the oil's characteristics, aromatherapy has been recognized to have antibacterial, wound-healing, immune enhancing, antidepressant, and calming curative effects [20]. Studies on the clinical effects of aromatherapy have shown that it alleviates stress and increases sleep quality. This includes studies on stress responses and sleep in hospitalized elderly patients [1], sleep and pain in cancer patients [21], sleep in children with autism, and quality of sleep in heart disease patients of hospital [22, 23].

Studies on ICU patients have examined the effects of aromatherapy on sleep in heart disease patients [24], high blood pressure [11], stress, and anxiety. However, these studies [11, 24] examined patients who had undergone treatment for only 1 day and took place only in coronary care units. Studies that examine stress and sleep quality in ICU patients for more than 2 days are lacking. Therefore, this study examined ICU patients who underwent aromatherapy treatment for more than 2 days and verified its effects of alleviating stress and improving sleep quality.

The explicit purposes of this study are to investigate the effect of aromatherapy on ICU patients' stress, blood pressure, heart rate, and sleep quality.

The research hypotheses were as follows.

*(1) Primary Hypothesis*. There will be significant differences in stress between the experimental group that undergoes aromatherapy treatment and the control group that does not.

*(2) Secondary Hypothesis*. There will be significant differences in quality of sleep between the experimental group and the control group.

## 2. Methods

### 2.1. Study Design

**Figure 1:**
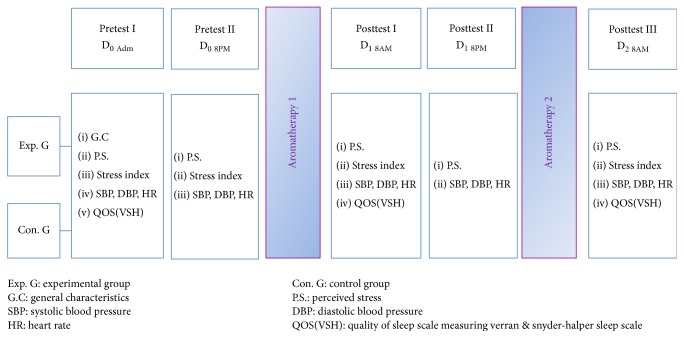
Study design.

This was an experimental study that used a nonrandomised pre- and posttest design that compared stress and sleep quality between 2 groups of ICU patients: those who underwent aromatherapy and those who did not. To prevent diffusion and contamination in the experimental treatment between the 2 groups, data were first collected from the control group (*n* = 32) from July to August 2016 and then from the experimental group (*n* = 32) from September to October 2016 (Figure 1).

### 2.2. Study Setting

This study was conducted at the intensive care unit in the university hospital. Participants included lucid adult patients who were admitted to the intensive care unit and had spent more than two nights there.

### 2.3. Participants

#### 2.3.1. Participant Selection

This study was conducted for ICU patients who had been admitted via emergency room, hospitalized in ICU of E University Hospital located in D city, for more than 2 nights from July 1st, 2016, to October 15th, 2016. This study was conducted under the consent of a doctor of pulmonology for patients suffering from asthma or chronic obstructive pulmonary disease.

Participants who met the following inclusion criteria were selected as participants: age > 18 and <70 years, being lucid and fully capable of communicating, being in the ICU for >2 nights, understanding the study's purpose, being permitted to participate in the study, and providing written consent. For patients aged > 60 years, a guardian provided written consent when applicable. Participants were excluded if they met the following criteria: having a mental illness, taking antianxiety or sleep aid medication, having side effects or allergies to aroma essential oils, being admitted to the ICU between 7 p.m. and 5 a.m. to make the subject's condition the same, having a systolic blood pressure below 100 mmHg (lavender essential oil can lower blood pressure), and having an arrhythmia.

#### 2.3.2. Sample Size Calculation

**Figure 2:**
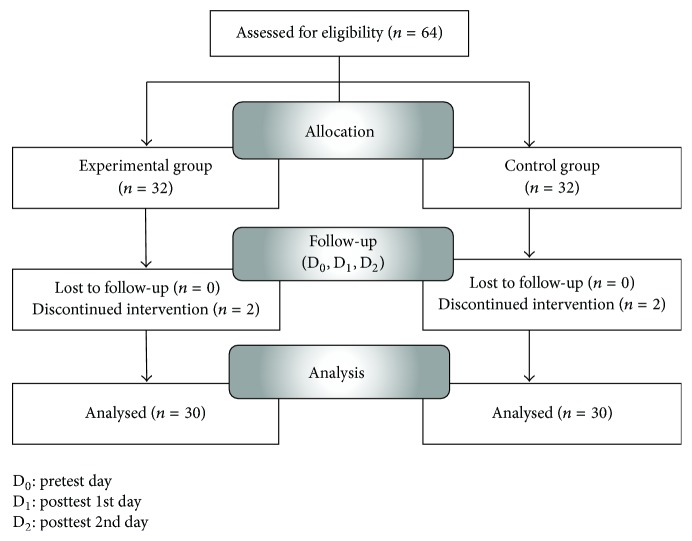
Flow diagram.

We calculated sample size using G. Power Analysis [25]. Assuming a significance level (*α*) of .05 statistical power (1 − *β*) of .80, and an effect size of 0.75 calculated in earlier studies [26], we sought to include 29 participants in each group. Data were collected from 32 participants in each group considering a 10% dropout rate. Excluding 2 participants with arrhythmia, 1 participant who refused to participate during the experiment, and 1 participant who was moved to another ward, 30 participants in each group were included in the final analysis (Figure 2).

### 2.4. Experimental Treatment

The experimental treatment in this study required patients who had spent more than 2 nights in the ICU to undergo aromatherapy before and during sleep.

#### 2.4.1. Selection and Storage of Aroma Oils

A prescription was received from one of the researchers with an aromatherapy certificate, which had a calming effect when it was used on a predetermined neural network. Lavender [27] was the first essential oil chosen for treating sleeplessness, and it was stored in a refrigerator when not being used.

#### 2.4.2. Application of Aromatherapy

- The experimental treatment was aromatherapy, which was applied within 1 hour of hospitalization and at 8 p.m. after the pretest, prior to the experiment. Perceived and objective stress, blood pressure, heart rate, and average sleep quality were measured within 1 hour of admittance to the ICU. At 8 p.m. on the same day, perceived and objective stress, blood pressure, and heart rate were measured. The experimental treatment began at 9 p.m. after a pretest, which was administered at 8 p.m. Three drops of lavender essential oil were applied to an aromastone and patients were directed to breathe deeply 10 times.
- After the oils were inhaled through deep breathing, an aromastone was hung on the centre of the bedside railing within 10 cm and participants were instructed to go to sleep (Aromatherapy 1).
- The aromastone was removed at 8 a.m. after participants had inhaled the oils and finished sleeping.
- On the second day of hospitalization, perceived stress, blood pressure, and heart rate were measured in the ICU; then, the experimental group was given an aromastone with 3 drops of lavender essential oil on it and instructed to breathe deeply 10 times.
- After the oils were inhaled through deep breathing, an aromastone was hung on the centre of the bedside railing within 10 cm, and participants were instructed to go to sleep (Aromatherapy 2).
- The aromastone was removed at 8 a.m. after participants had inhaled the oils and finished sleeping.

### 2.5. Measures

#### 2.5.1. Stress Measurements

*(i) Perceived Stress Using a Numeric Rating Scale*. The scale ranged from 0* (no stress at all)* to 10* (severe stress)*; participants self-reported felt stress.

*(ii) Stress Index*. The stress index is a value that quantifies stress levels with standard derivations. This measurement was based on heart rate variability, which was measured with Canopy9 professional 4.0 (IEMBIO, USA), an automatic nervous system measuring device. This index measures miniscule changes in periodic heart rate on a scale of 1–10. 

*(iii) Blood Pressure*. The Philips Intellivue MP50 patient monitor was used to measure blood pressure in participants. The monitoring system was placed on the upper arms of the patients at the same height as the heart, and the lower cuff covered 2 cm of the elbow. Systolic and diastolic blood pressure were then measured and expressed as mmHg units according to the measurement guidelines of the automatic sphygmomanometer. 

*(iv) Heart Rate*. The Philips Intellivue MP50 patient monitoring system was used to measure heart rate. After participants rested, heart rate was recorded and expressed as beats/minute.

#### 2.5.2. Sleep Quality Measurements

We used scores from the Verran & Snyder-Halpern Sleep Scale, which measured sleep quality over the course of 2 days at 8 a.m. when patients awoke. Kim and Kang [28] translated this tool. The scale comprises 9 questions including the number of times one wakes up during the night, how much one tosses and turns, total hours spent sleeping, the depth of sleep, how long it takes to fall asleep, how one feels when waking up, how one awakes from sleep, and level of satisfaction with sleep. The final question is a short-answer question. Excluding the short-answer question, the remaining 8 questions ranged from 0 to 10 points with a lowest possible score of 0 points and highest possible score of 80 points. Higher scores indicated higher quality of sleep. The reliability of this tool was measured using Cronbach's alpha: pretest, .87; first night of sleep, .97; second night of sleep, .98.

### 2.6. Data Collection

1. Data collection was conducted after explaining the collection procedures to the hospital's nurses and relevant departments, requesting cooperation, and receiving permission. The purpose of the study was explained to those who met the inclusion criteria, and data were collected from participants who agreed to participate.
2. Data were first collected from the control group to avoid diffusion of the experimental treatment.
3. Room instructions, room arrangements, the type of patient monitoring system, and its uses were all identical for patients being admitted. The researcher read the questionnaire and recorded the answers during the survey and checked whether a cannula was inserted or not for stability of patients.
4. All participants were hospitalized by way of the emergency room, and no additional tests were conducted after the initial inspection. The treatment patients received and their environment in the ICU were identical. Blood pressure and heart rate measurements were taken every hour in identical intervals, and the number of patient monitoring devices was identical for each patient.

### 2.7. Data Analysis

The collected data were analysed with using SPSS 23.0. Participants' general characteristics were analysed as percentages and means. The homogeneity of general characteristics between the experimental group and control group was analysed with a chi-square test and a *t*-test. A *t*-test, repeated measures analysis of variance (ANOVA), and analysis of covariance were used to analyse differences in perceived stress, the stress index, blood pressure, heart rate, and sleep quality before and after the experimental treatment in both groups. Before we analysed the data with repeated measures ANOVA, we tested whether the sphericity assumption was satisfied. If the assumption of sphericity assumption was not satisfied, we performed multivariate analysis. The reliability of the tool used to measure sleep quality was analysed with Cronbach's *α*.

### 2.8. Ethical Considerations

1. Approval was granted after submitting a research plan to E University Hospital's Institutional Review Board (EMC 2016-01-003-004).
2. Patients who had spent more than 2 nights in E University Hospital's ICU were selected as participants, and a pulmonologist's consent was received for any patients who were using a respirator. The experiment was conducted after explaining the study's purpose and methodology and receiving consent from the nurses and relevant medical teams.
3. Participation was voluntary; before agreeing to participate, participants and guardians were provided with a thorough explanation of the study.
4. The explanation form included information about the participants' right to participate or withdraw from the experiment, potential side effects and treatment methods for such side effects, and compensation.
5. To protect personal information, a serial number-based identification was provided based on guidelines for personal information processing.
6. As a gesture of gratitude, a small gift was provided to the participants after the data were collected.
7. This study was conducted in accordance with the Declaration of Helsinki.

## 3. Results

This study included a total of 60 subjects, 30 of whom were placed in an experimental group and 30 of whom were placed in a control group. The groups were then used to test the effects of aromatherapy on stress and sleep quality in ICU patients.

### 3.1. Homogeneity Test

The results of a homogeneity test regarding questions on demographic elements and health are depicted in Table 1. There was no significant difference between groups in the demographic element and health related characteristics of the subjects.

The prehomogeneity test results concerning the dependent variables are depicted in Table 1.

There were no significant differences between groups in initial systolic or diastolic blood pressure readings, heart rate, or perceived sleep quality. Regarding perceived stress and the objective stress index, the experimental group was significantly different than the control group.

In scores measuring perceived stress, the experimental group and control group had scores of 8.10 and 6.27, respectively, showing a significant difference between the groups (*t* = −4.294, *p* < .001). In the objective stress index, the experimental group scored 7.73, while the control group scored 6.17, again showing a significant difference between the groups (*t* = −2.396, *p* = .020).

In review, the prehomogeneity test results on dependent variables between the groups showed no significant differences in systolic blood pressure, diastolic blood pressure, heart rate, sleep quality scores, and attained homogeneity. Test results, however, did show a significant difference between groups in the perceived stress and objective stress index (Table 1).

### 3.2. The Effect of Aromatherapy on Stress and Vital Signs in ICU Patients

The measurement results over two days to verify the effect of aromatherapy on perceived stress are shown in Table 2. In an initial homogeneity test, perceived stress (D_0  Adm_), which showed a significant difference, was processed as a covariate. The results of this showed that, on the first morning (D_1  8am_) when patients had awoken after undergoing aromatherapy the previous night, perceived stress in the experimental group was 5.33 and 7.80 in the control group (*F* = 120.481, *p* < .001). Before going to sleep the same evening (D_1  8pm_), the experimental group had a score of 5.47 and the control group had a score of 8.23 (*F* = 167.557, *p* < .001) in perceived stress. On the second morning after undergoing aromatherapy the previous night (D_2  8am_), the experimental group had a score of 3.73 and the control group had a score of 8.50 in perceived stress, depicting a significant difference between the groups (*F* = 390.022, *p* < .001). The results of repeated measurements of perceived stress over the course of 2 days after processing perceived stress (D_0  Adm_) as a covariate showed that there was a significant difference based on time (*F* = 2.99, *p* = .033). There was also a significant difference between the two groups in their respective scores for perceived stress (*F* = 148.43, *p* < .001). Lastly, there was a significant difference in the results of the reciprocal interaction of time based on the group (*F* = 60.11, *p* < .001) (Table 2).

The measurement results over two days to verify the effect of aromatherapy on objective stress index are shown in Table 2. In a homogeneity test, the objective stress index (D_0  Adm_) was processed as a covariate. The results of this showed that, on the first morning (D_1  8am_), the experimental group and control group had objective stress index scores of 3.90 and 7.60 (*F* = 44.866, *p* < .001), respectively. Measurements on the second morning showed an objective stress score of 4.37 in the experimental group and 8.00 in the control group. These results show a significant difference between the groups (*F* = 74.309, *p* < .001). The results of repeated measurements of objective stress index over the course of two days after processing objective stress before the experimental treatment (D_0  Adm_) as a covariate are as follows. There was a significant difference in the results of the reciprocal interaction of time based on the group (*F* = 25.65, *p* < .001) (Table 2).

The measurement results over the course of two days to verify the effects of aromatherapy on blood pressure in the experimental group and control group are shown in Table 3. The results of repeated measures of ANOVA on blood pressure showed that on the first morning (D_1  8am_) blood pressure in the experimental group and control group was 116/71 mmHg and 135/74 mmHg, respectively. On the same evening before patients went to sleep, blood pressure in the experimental group was 119/70 mmHg and 129/71 mmHg in the control group. In the morning of the second day (D_2  8am_), blood pressure in the experimental group was 113/68 mmHg and 134/72 mmHg in the control group. The results of repeated measurements of systolic blood pressure over the course of two days showed that there was a significant difference based on time (*F* = 13.53, *p* < .001) and a significant difference in blood pressure scores between the two groups (*F* = 10.51, *p* = .002). The results of the reciprocal interaction with time based on group also showed a significant difference (*F* = 9.09, *p* < .001) (Table 3). The results of repeated measures of ANOVA on diastolic blood pressure over the course of two days showed that there was a significant difference based on time (*F* = 7.35, *p* < .001) and the reciprocal interaction of group and time (*F* = 2.47, *p* = .046). However, there was no significant difference between the diastolic blood pressure results between the groups (*F* = 0.076, *p* = .783) (Table 3).

The changes in heart rate measurements between both groups are shown in Table 3. There was a significant difference based on time and group. There was also a significant difference in the results of reciprocal interaction of time based on the group (*F* = 5.71, *p* < .001) (Table 3).

### 3.3. The Effect of Aromatherapy on Sleep Quality in ICU Patients

The Verran & Snyder-Halper (VSH) measurement tool was used in order to verify the effect of aromatherapy on sleep quality. Measurements were taken within 1 hour (D_0  Adm_) of hospitalization, on the first morning (D_1  8am_) after patients had awoken, and on the second morning after patients had awoken (D_2  8am_). The sleep quality of the experimental group and the control group within 1 hour (D_0  Adm_) of hospitalization had scores of 65.13 and 61.03, respectively (*t* = −0.640, *p* = .524). Sleep quality measurements were taken again on the first morning (D_1  8am_); sleep quality scores were 52.90 for the experimental group and 33.56 for the control group (*t* = −11.638, *p* < .001). Sleep quality on the second morning after patients had awoken (D_2  8am_) was 57.73 points for the experimental group and 25.80 points for the control group (*t* = −19.577, *p* < .001). The results of the repeated measure of ANOVA on sleep quality over the course of two days showed that there was a significant difference based on time (*F* = 294.60, *p* < .001) and that there was a significant difference in results based on the group (*F* = 221.12, *p* < .001). There was also a significant difference between the group and reciprocal interaction of time (*F* = 109.46, *p* < .001) (Table 2).

## 4. Discussion

This study investigated the effect of aromatherapy on stress and sleep quality in patients who had been admitted to the ICU and were being treated there. The experimental group consisted of participants (*n* = 30) breathing in lavender essential oil, which began after conducting a pretest.

### 4.1. Aromatherapy and Stress

Perceived stress and objective stress index were measured before and after the aromatherapy treatment to verify the effect of aromatherapy on stress. The results showed that perceived stress and the objective stress index in the experimental group that received aromatherapy decreased on both the first and second days. Conversely, perceived stress and the objective stress index in the control group increased on both the first and second days. Stress typically increases in patients when they are admitted to the ICU; however, stress decreased in the experimental group. This result demonstrates the antistress effect of lavender. This study verified that lavender aromatherapy is a significantly effective method for alleviating perceived and objective stress in ICU patients. This result is in line with preceding research that claims aromatherapy is effective in alleviating stress in heart disease patients [29]. The results of the perceived level of stress and objective stress index measured as heart rate variability in patients were identical, and the objective stress index was an indicator of perceived stress.

### 4.2. Aromatherapy and Blood Pressure

Systolic blood pressure decreased in both the experimental and control group between the time of admittance to the ICU and before bedtime at 8 p.m. This may reflect the initial stress of hospitalization, which caused systolic blood pressure to increase, and the effect of half a day's bedrest, which decreased stress and lowered systolic blood pressure. The experimental group used lavender and exhibited decreased systolic blood pressure on the first and second mornings. Conversely, the control group experienced an increase in blood pressure during this same period.

In addition, systolic blood pressure in the experimental group increased before bedtime without the lavender; however, it decreased by roughly 10 mmHg after using the lavender in the morning. Blood pressure typically undergoes periodic changes, and there is a tendency for blood pressure to rise due to a morning surge [30]. Therefore, a rise in blood pressure reflects the morning surge that occurs before 8 a.m. The experimental group, however, exhibited low blood pressure readings in the morning, which may demonstrate the effect of lavender in lowering blood pressure. These results show that aromatherapy using lavender is effective and is consistent with preceding research showing that systolic blood pressure drops after inhaling aroma essential oils [11].

As stress in the experimental and control groups decreased after hospitalization, diastolic blood pressure also decreased after a half day's rest. There was no dramatic change in diastolic blood pressure on the first and second days of the experiment that followed. These results are similar to previous results where systolic and diastolic blood pressure decreased in concert [11, 31]. This may show that autonomic arousal is suppressed with the use of lavender [26].

### 4.3. Aromatherapy and Heart Rate

Both groups exhibited a decrease in heart rate over time, which may be a result of resting their bodies after admittance to the ICU. The experimental group showed a continuous decrease in heart rate on the first and second days after the application of aromatherapy. These results also show the effectiveness of aroma inhalation.

### 4.4. Aromatherapy and Sleep Quality

Subjective sleep quality scores were measured to verify the effect of aromatherapy on sleep quality. The intensive care unit has various external stimuli such as consciousness evaluation, blood pressure measurement, and blood sampling. Therefore, in this study, objective sleep quality was not measured and subjective sleep quality was measured. It is very meaningful to measure the quality of sleep that the patient feels during the period of admission treatment in ICU.

The results of this study show that there was no difference between groups in the pretest, which examined average sleep quality; however, sleep quality decreased in both groups following hospitalization. This is likely a natural result of the hospitalization itself affecting sleep quality. Sleep quality in the control group dropped by 50% in both the first and second nights of hospitalization. Sleep quality in the experimental group, however, decreased only by roughly 10% after the experimental treatment. This indicates that lavender prevents extensive reductions in sleep quality in hospitalized patients and is consistent with preceding research that demonstrates lavender's effectiveness in promoting sleep quality [28].

### 4.5. Strengths

This study has three meaningful strengths:

- We verified the effect of aromatherapy on stress and sleep quality in ICU patients from a variety of aspects.
- We used aromatherapy to measure and verify its effect on stress and sleep quality throughout patients' treatment, rather than just measuring its effects on day-to-day stress and sleep quality.
- We presented subjective and objective data when measuring stress in patients.

### 4.6. Limitations

This study has some limitations. First, we collected data from the control group first to prevent diffusion and contamination of the experimental treatment. As such, the study could be affected by a difference in data collection periods. In addition, it was difficult to completely hide the information regarding which group the subject belongs to because of the aroma-like nature of the experimental procedure. Lastly, the intervention was only performed at one hospital, which limits the generalizability of the results. More research is necessary on whether aromatherapy is effective throughout the treatment process for ICU patients required to undergo long-term treatment. There also needs to be further studies on stress relief and improvements in sleep quality in both ICU patients and patients who are moved to other wards.

## 5. Conclusion

This experimental study used a nonrandomised, pre- and posttest design to address the effect of aromatherapy on stress and sleep quality among ICU patients. The results showed significant differences in perceived stress, objective stress index, blood pressure, heart rate, and sleep quality between the experimental group who received aromatherapy treatment and the control group who did not. Overall, the above research results demonstrate that aromatherapy, which was administered over two days, reduced stress and improved sleep quality in ICU patients.

## Figures and Tables

**Table 1:** Homogeneity test of baseline for participants (*N* = 60).

Characteristics	Category	Exp. (*n* = 30)	Cont. (*n* = 30)	*x* ^2^ or *t*	*p*
		*N* (%)	*N* (%)		
Sex	Male	17 (56.7%)	15 (50.0%)	0.268	.605
	Female	13 (43.3%)	15 (50.0%)		
Age		59.50 ± 9.10	61.53 ± 8.80	0.880	.383
Hospital admission	No	11 (36.7%)	7 (23.3%)	1.270	.260
	Yes	19 (63.3%)	23 (76.7%)		
ICU admission	No	24 (80.0%)	22 (73.3%)	0.373	.542
	Yes	6 (20.0%)	8 (26.7%)		
Diagnosis (problem)	Internal medicine	23 (76.6%)	23 (76.6%)	0.000	1.000
	Surgery	7 (23.4%)	7 (23.4%)		
WMSCN^*∗*^	Group 4	20 (66.6%)	19 (63.4%)	0.266	.791
	Group 5	10 (33.4%)	11 (36.6%)		
NPO	No	13 (43.3%)	9 (30.0%)	1.148	.284
	Yes	17 (56.7%)	21 (70.0%)		
IV route	Peripheral route	23 (76.7%)	20 (66.7%)	0.739	.390
	Central route	7 (23.3%)	10 (33.3%)		
Cannula state	1 cannula	27 (90.0%)	23 (77.6%)	1.385	.172
	2 or more cannulas	3 (10.0%)	7 (13.4%)		
Perceived stress (D0_Adm_)		8.10 ± 1.61	6.27 ± 1.70	−4.294	<.001
Stress index (D0_Adm_)		7.73 ± 1.78	6.17 ± 3.11	−2.396	.020
BP (D0_Adm_)	SBP	134.60 ± 16.91	137.87 ± 20.82	0.667	.507
	DBP	82.00 ± 21.23	75.67 ± 13.65	−1.375	.175
HR (D0_Adm_)		92.20 ± 19.53	92.07 ± 20.95	−0.025	.980
Quality of sleep (D0_Adm_)		65.13 ± 5.70	64.03 ± 7.84	−0.622	.536

^*∗*^WMSCN: Workload Management System for Critical Care Nurses; Group 4: intensive care group; Group 5: continuous care group.

**Table 2:** The effects of aromatherapy on stress and sleep quality.

		Exp. (*n* = 30)	Cont. (*n* = 30)	*t* or *F*	*p*	*F* (*p*)
		M ± SD	M ± SD			
Perceived stress	Pretest 1 (D_0 Adm_)	8.10 ± 1.61	6.27 ± 1.70	−4.294	<.001	*Time* 2.99 (*p* = .033) *Group* 148.43 (*p* < .001) *Group∗Time* 60.11 (*p* < .001)
	Pretest 2 (D_0 8PM_)	8.23 ± 2.03	7.27 ± 1.70	0.129	.721	
	Posttest 1 (D_1 8AM_)	5.33 ± 1.24	7.80 ± 1.24	120.481	<.001	
	Posttest 2 (D_1 8PM_)	5.47 ± 0.97	8.23 ± 1.04	167.557	<.001	
	Posttest 3 (D_2 8AM_)	3.73 ± 0.87	8.50 ± 0.90	390.022	<.001	

Stress index	Pretest 1 (D_0 Adm_)	7.73 ± 1.79	6.17 ± 3.11	−2.396	.020	*Time* 0.52 (*p* = .597) *Group* 52.91 (*p* < .001) *Time∗Group* 25.65 (*p* < .001)
	Pretest 2 (D_0 8PM_)	8.37 ± 1.79	7.80 ± 2.66	0.382	.539	
	Posttest 1 (D_1 8AM_)	3.90 ± 2.23	7.60 ± 2.70	44.866	<.001	
	Posttest 2 (D_2 8AM_)	4.37 ± 1.97	8.00 ± 1.89	74.309	<.001	

Quality of sleep	Pretest 1 (D_0 Adm_)	65.13 ± 5.69	61.03 ± 7.83	−.640	.524	*Time* 294.60 (*p* < .001) *Group* 221.12 (*p* < .001) *Time∗Group* 109.46 (*p* < .001)
	Posttest 1 (D_1 8AM_)	52.90 ± 5.57	33.56 ± 7.15	−11.638	<.001	
	Posttest 2 (D_2 8AM_)	57.73 ± 5.61	25.80 ± 6.95	−19.577	<.001	

Exp. = experimental group; Cont. = control group; M ± SD: mean ± standard deviation; D_0  Adm_: admission day; D_0  8PM_: admission day before sleep; D_1  8AM_: experimental 1st day, 8 a.m.; D_1  8PM_: experimental 1st day, 8 p.m.; D_2  8AM_: experimental 2nd day, 8 a.m.

**Table 3:** The effects of aromatherapy on blood pressure and heart rate.

		Exp. (*n* = 30)	Cont. (*n* = 30)	*t* or* F*	*p*	*F* (*p*)
		M ± SD	M ± SD			
SBP (mmHg)	Pretest 1 (D_0 Adm_)	134.60 ± 16.90	137.87 ± 20.81	0.667	.507	*Time* 13.53 (*p* < .001) *Group* 10.51 (*p* = .002) *Time∗Group* 9.09 (*p* < .001)
	Pretest 2 (D_0 8PM_)	125.70 ± 13.56	127.63 ± 20.58	0.430	.669	
	Posttest 1 (D_1 8AM_)	116.37 ± 10.82	134.87 ± 17.53	4.917	<.001	
	Posttest 2 (D_1 8PM_)	119.27 ± 13.66	128.77 ± 19.46	2.188	.033	
	Posttest 3 (D_2 8AM_)	112.53 ± 9.47	133.53 ± 16.17	6.136	<.001	

DBP (mmHg)	Pretest 1 (D_0 Adm_)	82.00 ± 21.23	75.67 ± 13.64	−1.38	.175	*Time* 7.35 (*p* < .001) *Group* .076 (*p* = .783) *Time∗Group* 2.47 (*p* = .046)
	Pretest 2 (D_0 8PM_)	71.80 ± 12.69	73.70 ± 13.02	0.57	.569	
	Posttest 1 (D_1 8AM_)	71.17 ± 12.06	73.73 ± 12.70	0.80	.426	
	Posttest 2 (D_1 8PM_)	69.77 ± 11.49	70.47 ± 11.06	0.24	.811	
	Posttest 3 (D_2 8AM_)	67.60 ± 9.04	72.23 ± 13.51	1.56	.124	

HR (beats/min)	Pretest 1 (D_0 Adm_)	92.20 ± 19.52	92.07 ± 20.95	−.025	.980	*Time* 20.32 (*p* < .001) *Group* 6.23 (*p* = .015) *Time∗Group* 5.71 (*p* < .001)
	Pretest 2 (D_0 8PM_)	82.63 ± 15.56	93.33 ± 23.26	2.094	.041	
	Posttest 1 (D_1 8AM_)	74.93 ± 14.42	85.80 ± 21.19	2.321	.024	
	Posttest 2 (D_1 8PM_)	72.20 ± 11.83	88.90 ± 22.44	3.605	<.001	
	Posttest 3 (D_2 8AM_)	71.97 ± 11.84	84.73 ± 16.47	3.446	<.001	

Exp. = experimental group; Cont. = control group; M ± SD: mean ± standard deviation; D_0  Adm_: admission day; D_0  8PM_: admission day before sleep; D_1  8AM_: experimental 1st day, 8 a.m.; D_1  8PM_: experimental 1st day, 8 p.m.; D_2  8AM_: experimental 2nd day, 8 a.m.
